# Genomic population structure of Striped Bass (*Morone saxatilis*) from the Gulf of St. Lawrence to Cape Fear River

**DOI:** 10.1111/eva.12990

**Published:** 2020-05-29

**Authors:** Nathalie M. LeBlanc, Benjamin I. Gahagan, Samuel N. Andrews, Trevor S. Avery, Gregory N. Puncher, Benjamin J. Reading, Colin F. Buhariwalla, R. Allen Curry, Andrew R. Whiteley, Scott A. Pavey

**Affiliations:** ^1^ Department of Biological Sciences Canadian Rivers Institute University of New Brunswick Saint John NB Canada; ^2^ Massachusetts Division of Marine Fisheries Annisquam River Marine Fisheries Station Gloucester MA USA; ^3^ Department of Biology Canadian Rivers Institute University of New Brunswick Fredericton NB Canada; ^4^ Departments of Biology and Mathematics & Statistics Acadia University Wolfville NS Canada; ^5^ Molecular Biology Laboratory Maurice Lamontagne Institute, Fisheries and Oceans Canada Mont‐Joli QC Canada; ^6^ Department of Applied Ecology North Carolina State University Raleigh NC USA; ^7^ Pamlico Aquaculture Field Laboratory North Carolina State University Aurora NC USA; ^8^ Inland Fisheries Division Nova Scotia Fisheries and Aquaculture Pictou NS Canada; ^9^ Department of Biology and Faculty of Forestry and Environmental Management Canadian Rivers Institute University of New Brunswick Fredericton NB Canada; ^10^ Department of Ecosystem and Conservation Sciences and Wildlife Biology Program W. A. Franke College of Forestry and Conservation University of Montana Missoula MT USA

**Keywords:** aquatic ecology, candidate gene identification, ecology, population genetics, population genomics

## Abstract

Striped Bass, *Morone saxatilis* (Walbaum, 1792), is an anadromous fish species that supports fisheries throughout North America and is native to the North American Atlantic Coast. Due to long coastal migrations that span multiple jurisdictions, a detailed understanding of population genomics is required to untangle demographic patterns, understand local adaptation, and characterize population movements. This study used 1,256 single nucleotide polymorphism (SNP) loci to investigate genetic structure of 477 Striped Bass sampled from 15 locations spanning the North American Atlantic coast from the Gulf of St. Lawrence, Canada, to the Cape Fear River, United States. We found striking differences in neutral divergence among Canadian sites, which were isolated from each other and US populations, compared with US populations that were much less isolated. Our SNP dataset was able to assign 99% of Striped Bass back to six reporting groups, a 39% improvement over previous genetic markers. Using this method, we found (a) evidence of admixture within Saint John River, indicating that migrants from the United States and from Shubenacadie River occasionally spawn in the Saint John River; (b) Striped Bass collected in the Mira River, Cape Breton, Canada, were found to be of both Miramichi River and US origin; (c) juveniles in the newly restored Kennebec River population had small and nonsignificant differences from the Hudson River; and (d) tributaries within the Chesapeake Bay showed a mixture of homogeny and small differences among each other. This study introduces new hypotheses about the dynamic zoogeography of Striped Bass at its northern range and has important implications for the local and international management of this species.

## 1 INTRODUCTION

**FIGURE 1:**
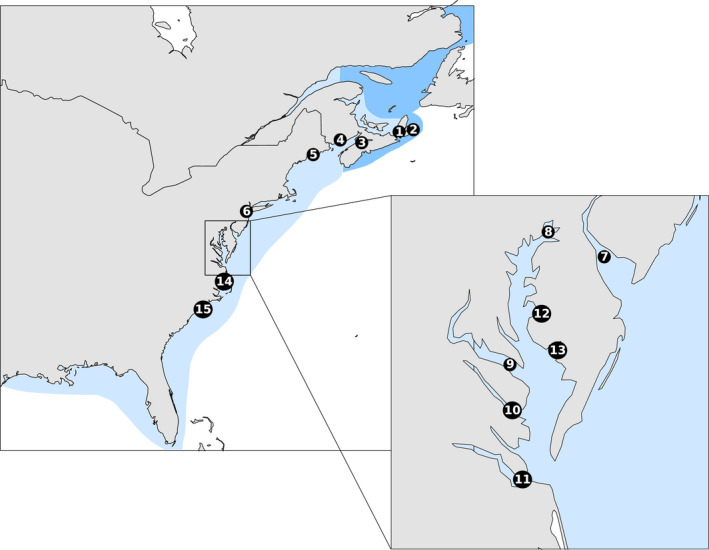
On the left, a map showing the current range of Striped Bass along the North American Atlantic Coast. Potential additions to the range where Striped Bass have been reported in or may inhabit are marked in darker green. Sampling sites marked in numbered circles as follows and listed according to the location of the river mouth. (1) Bras d’Or Lake, Nova Scotia; (2) Mira River, Nova Scotia; (3) Shubenacadie River, Nova Scotia; (4) Saint John River, New Brunswick; (5) Kennebec River, Maine; (6) Hudson River, New York/New Jersey; (14) Roanoke River, North Carolina; and (15) Cape Fear River, North Carolina. On the right, a close up of Delaware River and Chesapeake Bay, with sampling locations marked as follows. (7) Delaware River, New Jersey/Delaware; (8) upper Chesapeake Bay, Maryland; (9) Potomac River, Maryland; (10) Rappahannock River, Virginia; (11) James River, Virginia; (12) Choptank River, Maryland; and (13) Nanticoke River, Maryland

The Striped Bass, *Morone saxatilis* (Walbaum, 1792), is a facultative anadromous and economically important fish with a native range extending along the Atlantic coast of North America from the St. Lawrence River, Quebec, to the St John's River, Florida, as well as a native population in the Apalachicola–Chattahoochee–Flint river system in the Gulf of Mexico (Setzler et al., 1980; Wirgin, Currie, Roy, Maceda, & Waldman, 2005; Figure 1). Individuals from the Hudson River to the Roanoke River can move long distances, some moving 400–1000 km along the Atlantic Coast (Callihan, Harris, & Hightower, 2015; Kneebone, Hoffman, Dean, Fox, & Armstrong, 2014; Mather et al., 2010), and along with some Canadian populations are known to enter non‐natal rivers (Grothues, Able, Carter, & Arienti, 2009; Kneebone et al., 2014; LeBlanc et al., 2018). Migratory populations within the United States are currently managed as two separate stocks: the Roanoke River, and all US populations north of the Roanoke River (Atlantic States Marine Fisheries Commission (ASMFC, 2019)). Populations south of the Roanoke River and Albemarle Sound are generally considered nonmigratory (Bjorgo, Isely, & Thomason, 2000), Striped Bass in the Gulf of St. Lawrence are thought to be isolated from the rest of the range (Rulifson & Dadswell, 1995), and it is unknown whether Bay of Fundy populations travel further than the Gulf of Maine (Department of Fisheries and Oceans (DFO), 2014). Striped Bass throughout its range experienced severe population declines from the 1960s to 1980s, leading to extensive temporary and permanent closures of commercial and recreational fisheries (Andrews, Dadswell, Buhariwalla, Linnansaari, & Curry, 2019; Carmichael, Haeseker, & Hightower, 1998; Richards & Rago, 1999). Multistate emergency management measures implemented by the ASMFC in the United States resulted in the recovery of most US populations during the 1990s and 2000s (ASMFC, 2019; Richards & Rago, 1999), although abundance estimates have since declined in the 2010s (ASMFC, 2019). In Canada, the closure of commercial fisheries and restrictions on recreational fishing led to the recovery of some populations (Miramichi River and Shubenacadie River) and not others (Saint John River, Annapolis River, and St. Lawrence River; Andrews, Linnansaari, Leblanc, Pavey, & Curry, 2019).

Effective management of a highly migratory species requires knowledge of the connectivity between populations and the seasonal mixing rates of multiorigin stocks in relation to spatial dynamics within the species range. Striped Bass populations have complex life histories and often exhibit multiple migratory components, or contingents (Andrews, Linnansaari, Curry, & Dadswell, 2017; Clark, 1968; Gahagan, Fox, & Secor, 2015; Secor, 1999; Secor, Rooker, Zlokovitz, & Zdanowicz, 2001), which appear tied to ontogenic development (Conroy, Piccoli, & Secor, 2015; Gahagan et al., 2015) and population size (Callihan, Godwin, & Buckel, 2014; Waldman, Dunning, Ross, & Mattson, 1990). Partial migration, where some individuals in a population are resident while others migrate, and contingent behaviors further complicate management of Striped Bass as harvest in coastal waters may be on mixed stocks from multiple populations and also from specific behavioral subsets of those populations. Coastal migrations can facilitate genetic exchange between populations because mixing away from natal rivers may lead to some individuals straying and spawning in non‐natal rivers, an infrequent yet measurable occurrence (e.g., Gauthier et al., 2013; LeBlanc et al., 2018). In anadromous fishes, straying allows moving, expanding or contracting its range in response to environmental changes (Pess, Quinn, Gephard, & Saunders, 2014). Striped Bass inhabiting areas once covered by the Laurentide Ice Sheet, that is, the entirety of the Bay of Fundy and Gulf of St. Lawrence must have descended from southern migrants colonizing these rivers within the last 10,000 years as the glaciers retreated (Curry, 2007; Pielou, 1991).

Over the past five decades, Striped Bass stock discrimination has been attempted using many techniques producing inconsistent results when matching individuals to more than two reference populations (Waldman & Fabrizio, 1994; Waldman, Maceda, & Wirgin, 2012).The Chesapeake Bay is usually considered the primary source of migratory Striped Bass found along the North American Atlantic Coast, with the Hudson River occasionally providing large numbers and the Delaware and Roanoke Rivers previously considered to have a negligible contribution (Richards & Rago, 1999; Wirgin, Waldman, Maceda, Stabile, & Vecchio, 1997). Mixed‐stock analyses have found that stock composition can vary dramatically. Hudson River Striped Bass can contribute 14%–89% of coastal aggregations in different seasons and locations and from year to year (Fabrizio, 1987; Wirgin, Maceda, Waldman, & Crittenden, 1993). Existing mixed‐stock methods are often unable to reliably differentiate Roanoke River and Chesapeake Bay individuals, and consequently, the Roanoke River population is often merged with the Chesapeake Bay in reference groups, making it difficult to track relative contribution of Roanoke River Striped Bass to the current coastal groups (Waldman & Fabrizio, 1994; Waldman et al., 2012). The Delaware River is often not considered in coastal stocks, because the Delaware River population is small and was not expected to contribute to coastal aggregations in previous decades (Waldman & Fabrizio, 1994; Waldman et al., 2012); however, acoustic telemetry showed Delaware River Striped Bass make up 14%–20% of Striped Bass caught off the coast of Massachusetts (Kneebone et al., 2014). A mixed‐stock analysis that can reliably distinguish among stocks that exhibit varying degrees of mixing in the coastal environment could substantially improve Striped Bass management.

In addition to the ongoing attempts to characterize Striped Bass migration, the last decade has seen shifts in the existing range of several populations. Large‐sized Striped Bass in the Roanoke River population, previously considered largely resident because few tagged fish have been caught outside the river, have been recently shown to migrate approximately 500–600 km to New Jersey (Callihan et al., 2015). Striped Bass from the Miramichi River, which is considered the only spawning population in the Gulf of St. Lawrence (Robinson, Courtenay, Benfey, Maceda, & Wirgin, 2004), have been caught off the Labrador coast following a decade of strong population growth (Andrews, Dadswell, et al., 2019; DFO, 2018). These apparent range expansions have been attributed to increased ocean temperature (DFO, 2018), increased population size (Andrews, Dadswell, et al., 2019; Callihan et al., 2014), and an increase in the number of older, larger adults that are more likely to migrate longer distances (Callihan et al., 2014). These emerging migrations highlight the need to apply more sophisticated population discrimination tools to best inform management.

Several attempts have been made to use genetic markers in mixed‐stock analysis of Atlantic Coast Striped Bass (Brown, Baltazar, & Hamilton, 2005; Gauthier et al., 2013; Wirgin, Maceda, et al., 1993; Wirgin, Waldman, et al., 1997), within the Bay of Fundy (Wirgin et al., 1995), and the Gulf of St. Lawrence (Robinson et al., 2004). Two studies have comprehensively investigated the genetic structure among the major migratory populations of the North American Atlantic Coast (Gauthier et al., 2013; Wirgin, Maceda, Tozer, Stabile, & Waldman, 2020). Previous studies have found consistent genetic differences among known Canadian populations (Bentzen & Paterson, 2008; Wirgin, Ong, et al., 1993), and lower but significant differences between regions such as the Hudson River and Chesapeake Bay (Gauthier et al., 2013; Wirgin, Waldman, et al., 1997); however, rivers in close proximity to each other, particularly the Chesapeake Bay and Delaware River, have had inconsistent results (see Brown et al., 2005). Most recently, Gauthier et al. (2013) and Wirgin et al. (2020) found very low but significant differences among rivers within the Chesapeake Bay using 14 and 8 microsatellites, respectively, but both were unable to assign a high number of individuals to a river of origin.

Genotyping by sequencing (GBS) can be used to construct large panels of single nucleotide polymorphisms (SNPs) throughout the genome of an individual organism (Narum, Buerkle, Davey, Miller, & Hohenlohe, 2013; Poland, Brown, Sorrells, & Jannink, 2012). The SNP panels created by GBS can discriminate among closely related populations of anadromous fishes such as Alewife (*Alosa pseudoharengus*) and Blueback Herring (*A. aestivalis*; Baetscher, Hasselman, Reid, Palkovacs, & Garza, 2017) and Atlantic Salmon (*Salmo salar*; Bourret et al., 2013). Large numbers of SNPs also facilitate identification of genes or regions showing signs of selection, by examining which of the hundreds or thousands of SNPs show significantly greater differentiation among populations (Allendorf, Hohenlohe, & Luikart, 2010). While these outlier analyses are biased toward detection of single loci with strong signals of selection over more subtle polygenic adaptation (Rockman, 2012), they can serve as a starting point for identifying adaptive differences between populations. Moreover, inclusion of outlier loci in tests of population differentiation can disproportionately bias results (Allendorf & Seeb, 2000; Luikart, England, Tallmon, Jordan, & Tab erlet, 2003). Once identified, these loci can then be removed from analyses of genetic structure, migration, and effective population size, and examined separately to gain insights into adaptive selection that may be occurring in a population and highlight potential candidate genes for future studies.

In this work, we employ next‐generation sequencing to examine the genetics of Striped Bass from 14 locations across the native range, from the Gulf of St. Lawrence to the southernmost edge of the migratory range in the Roanoke River (Figure 1). We sample two locations (Hudson River and Delaware River) in two different years to assess temporal stability of populations. We include samples from six tributaries within the Chesapeake Bay to examine small‐scale spatial differences. Also included are samples from the Cape Fear River, which has a supportive breeding program to maintain a Striped Bass population in‐river, the recently restored Kennebec River, and from the Mira River on the northeastern coast of Nova Scotia, which is speculated to host a spawning aggregation of Striped Bass (Buhariwalla, 2018). We assess neutral genetic structure and characteristics of SNPs that show signs of selection, and we test the ability of our SNP dataset to assign Striped Bass back to their natal population.

## 2 METHODS

### 2.1 Sample collection

Fin clips and scales were taken from Striped Bass from multiple collections (Table 1). Age of sampled individuals differed by location. YOY juveniles were individuals less than 1 year old (<15 cm long). Saint John juveniles were 1–4 years old and largely spawned in the year 2013. Ages for Saint John River juveniles were obtained from scales. Adults were sexually mature individuals aged 4 years and older. All adults collected were in spawning condition at time of sampling, except for Bras d’Or Lake, Mira River, and Shubenacadie River. Shubenacadie origin Striped Bass migrate to the Stewiacke–Shubenacadie systems from overwintering sites during the sampling period (DFO, 2014; Keyser, Broome, Bradford, Sanderson, & Redden, 2016). Adult bass caught during this period are assumed to be of Shubenacadie River origin for the purpose of population surveys (DFO, 2014). Putative Miramichi River origin Striped Bass were included using fin clips taken from Striped Bass caught in the Bras d’Or Lake, Cape Breton, that have previously been examined using microsatellites and found to match the Miramichi River population (Bentzen, Mcbride, & Paterson, 2014). These samples will hereafter be referred to as Bras d’Or–Miramichi individuals (Box 1).

**TABLE 1:** Number, collection date, type of tissue, and age of fish for each of 15 locations Striped Bass samples were collected in

Location	*n*	Date collected	Type	Age
BD‐MICHI	19	June‐Nov 2012–2014	Fin Clips	Adults
MIRA	22	April‐June 2013–2017	Fin Clips	Adults
SHUB	33	2014–2017	Scales	Adults
SJR	32	July‐Sept 2014–2017	Fin clips	Juveniles
KEN	16	April‐May 2012	Fin clips	Juveniles (YOY)
HUD 2012	34	May, 2014	Fin clips	Adults, spawning condition
HUD 2014	21	April‐May, 2012	Fin clips	Adults, spawning condition
DEL 2012	28	April 2014	Fin clips	Adults, spawning condition
DEL 2014	29	April 2012	Fin clips	Adults, spawning condition
CHPK	27	July, Sept 2011	Fin clips	Adults, spawning condition
POT	33	April‐May 2014	Fin clips	Juveniles (YOY)
RAPP	32	April 2014	Fin clips	Adults, spawning condition
JAMES	33	August, Sept 2011	Fin clips	
CHOP	33	June, Dec 2011	Fin clips	Juveniles (YOY)
NANTI	33	April 2014	Fin clips	Juveniles (YOY)
ROA	30	April‐May 2015	Fin clips	Adults, spawning condition
CF	22	April‐May 2015	Fin clips	Adults, spawning condition

Abbreviations: BD‐MICHI, Bras d’Or–Miramichi; CF, Cape Fear; CHOP, Choptank River; CHPK, Upper Chesapeake Bay; DEL, Delaware River; HUD, Hudson River; JAMES, James River; KEN, Kennebec River; MIRA, Mira River; NANTI, Nanticoke River; POT, Potomac River; RAPP, Rappahannock River; ROA, Roanoke River; SHUB, Shubenacadie River; SJR, Saint John River.

BOX 1 The fields of ecology, evolution and conservation have been greatly enhanced due to the rapid development of genomic technologies. This change started around 2007 when two genomics technologies; (a) high‐resolution genotyping (acquiring thousands of genotypes that were comparable among individuals and populations); and (b) transcriptomic profiling (measuring gene expression of thousands of genes simultaneously); began to be widely applied to study wild populations in nature. Another important transition occurred in approximately 2010, which is when applications of DNA barcoding greatly expanded. Louis Bernatchez has always been on the forefront of these changes and applying them to nonmodel organisms, often in natural settings. Many of these species had few genomic tools developed and many of the datasets were “messy” in comparison with zebrafish in laboratory settings, requiring innovated data analysis strategies. Louis has always embraced these situations as the variability is not a nuisance but rather fundamental to the way nature works. Highly duplicated genomes, traits controlled by many loci, panmictic or nearly panmictic species, are just a few of the messy systems that Louis has studied which have resulted in substantial insights about how selection, drift, migration, and mutation affect Earth’s biodiversity.

### 2.2 Laboratory

DNA was isolated using either NucleoMag^®^ 96 Tissue (Macherey‐Nagel) kit on an epMotion 5075t (Cat. 5075000302), or the E.Z.N.A. Tissue DNA Kit (Omega Bio‐Tek). Libraries containing 96 individuals each were prepared using a double‐digest restriction‐site‐associated DNA sequencing (ddRAD‐seq or ddRAD) protocol developed by Poland et al. (2012) and modified as described in LeBlanc et al. (2018). Samples were randomized so that each lane contained individuals from multiple locations and sequenced using Illumina^®^ HiSeq™ 2,500 or Illumina^®^ HiSeq™ 4000 (San Diego) at Génome Québec Innovation Centre.

### 2.3 Quality control and analysis

SNPs were demultiplexed and filtered using modified versions of Eric Normandeau's Stacks workflow scripts, available on github (https://github.com/enorman deau/stacks_workflow, downloaded August 2016). Cutadapt v. 1.13 (Martin, 2011) was used to trim adapters from the raw sequences using a maximum error rate (*e*) of 0.2 and a minimum read length (m) of 50. FastQC v. 0.11.5 (Babraham Bioinformatics) was used to assess sequence quality before and after. Sequences were then trimmed to a uniform length of 85 bp and demultiplexed using the *process radtags* module of Stacks v. 1.46 (Catchen, Hohenlohe, Bassham, Amores, & Cresko, 2013) using the paired‐end option –P. BWA version 0.7.15 (Li & Durbin, 2010) was used to align sequences to the Striped Bass genome (BioProject accession number PRJNA266827) using a minimum seed length (*k*) of 19, a maximum seed occurrence of 55, and no filtering on output alignment score, and otherwise default parameters. The stacks module *pstacks* identified reference aligned loci with a minimum depth (m) of 4 using the “snp” model type and an alpha of .1. Loci were assembled into a catalogue using *cstacks*, *sstacks*, and *rxstacks* with default settings, and unclear or unlikely haplotypes, as well as SNPs with a log likelihood <45, were pruned from the dataset. Using the *populations* module, SNPs were further filtered to remove all loci with a stack depth <5, with >20% missing data in any given location, and any loci not amplified in all locations. We examined the output of populations and removed loci with an Fis < −0.3 to eliminate possible paralogs, and used VCFTools 0.1.13 (Danecek et al., 2011) to remove any loci with a minor allele frequency <0.01, and plink v. 1.90 (Chang et al., 2015) was used to remove loci in linkage disequilibrium with each other.

Structure files created by Stacks were converted to the appropriate input files for downstream analyses using PGDSpider v. 2.1.1.0 (Lischer & Excoffier, 2012). Sibship analyses were carried out in Colony2 v. 2.0.6.5 (Jones & Wang, 2010) on each population separately to ensure individuals were not closely related. Full sibling pairs identified with a probability of >.5 were removed from subsequent analyses. Percent polymorphism of loci in each population was reported by the Stacks *populations* module, and expected and observed heterozygosity were calculated using the R package *adegenet* v. 2.1.1 (Jombart, 2008).

An initial pairwise *F*_ST_ analysis was conducted in Arlequin v. 3.5.2.2 (Excoffier & Lischer, 2010), with significance assessed using 10,000 random permutation tests. Individuals caught in the Hudson River and Delaware River in 2012 and 2014 were grouped by location and year in order to assess whether the genetic profile of each location differed from year to year. After confirming no significant differences between years, the two sampling years were pooled together for outlier analyses.

### 2.4 Constructing a neutral SNP panel and assessing adaptive selection

Outlier loci were removed prior to subsequent population genetic analyses, and a subset of outliers were examined separately. Existing outlier analyses are known to detect high numbers of false positives alongside true outlier loci (De Villemereuil, Frichot, Bazin, François, & Gaggiotti, 2014; Lotterhos & Whitlock, 2014), and a common method of controlling for this is to examine which loci are flagged as having non‐neutral divergence patterns by more than one analysis software (De Villemereuil et al., 2014). In the absence of out‐group genotypes or known neutral loci, we assessed whether any of our SNPs were under balancing or divergent selection using two methods. BAYESCAN v. 2.1 (Foll & Gaggiotti, 2008) was run with 100,000 iterations, using a burn‐in of 50,000, a thinning interval of 10, and a sample size of 5 K. Prior odds were set to 1,000 to minimize false positives while retaining power to detect outliers (Lotterhos & Whitlock, 2014). We also used the recently developed R package OutFLANK (Whitlock & Lotterhos, 2015) with Hmin >0.1 to identify an additional set of outliers. Unlike previous outlier tests like BayeScan, outFLANK uses distribution of allele frequencies across all loci to account for differences in genetic structure among populations (Whitlock & Lotterhos, 2015). Loci identified as outliers at a *q*‐value ≤0.05 by either method were removed to create a dataset of putatively neutral loci for genetic structure analyses. Loci identified as outliers by both methods were mapped to one of 35,010 scaffolds contained in the published Striped Bass genome using the JBrowse genome browser (Skinner, Uzilov, Stein, Mungall, & Holmes, 2009) to identify associated genes showing signatures of selection, and allele frequencies were calculated in Arlequin to investigate divergence patterns across populations.

### 2.5 Connectivity of Striped Bass locations through population genetic structure

Population structure was assessed using both traditional *F*_ST_ and clustering algorithms. Overall hierarchal *F*_ST_ and pairwise *F*_ST_ were calculated in Arlequin, and significance was assessed using 10,000 random permutation tests. Hierarchal population groupings for overall *F*_ST_ were made based on patterns of differentiation seen in clustering analyses and previous studies. Pairwise *F*_ST_ values were also calculated on all locations, and pairwise significance was assessed using a chi‐square test implemented in the R package *strataG* v. 2.1 (Archer, Adams, & Schneiders, 2017), and corrected to account for multiple tests using the false discovery rate method detailed in Benjamini and Hochberg (1995). Chi‐square tests have high power and low false‐positive rates when used on large numbers of biallelic loci, as found in SNP datasets (Ryman et al., 2006).

Isolation by distance (IBD) was assessed using mantel tests implemented in Arlequin v. 3.5.2.2 (Excoffier & Lischer, 2010). Isolation by distance was assessed on all locations, on only Canadian locations, only US locations, and on locations within Chesapeake Bay and Delaware River, using approximate distance between rivers. When calculating distances between rivers, we assumed that Striped Bass make use of the Cape Cod Canal, and that Striped Bass move between the Chesapeake Bay and the Delaware River via the Chesapeake and Delaware Canal based upon tagging study results (Gahagan et al., 2015; Kneebone et al., 2014).

The R package LEA v. 2.0 (Frichot & François, 2015) estimates ancestry coefficients for all individuals using sparse non‐negative matrix factorization (sNMF), an algorithm that has been optimized for use with large numbers of genetic markers. Scenarios were run using 1–20 theorized number of distinct genetic populations (*K*), with 10 repetitions per *K* value, on all individuals as well as on only US individuals. To ensure results were not biased by differences in sampling size, sNMF was run a second time with a maximum of 30 individuals from any given genetic cluster found in the initial run. The probability of a K being valid was calculated using the cross‐entropy criterion. The K values with the lowest minimal cross‐entropy value were considered most probable as the true number of ancestral populations (Frichot, Mathieu, Trouillon, Bouchard, & François, 2014). Where the lowest entropy was unclear, clustering results for the lowest *K* values were manually inspected for informative grouping and consistency across repetitions. Population structure was also assessed using Discriminant Analysis of Principal Components (DAPC; Jombart, Devillard, & Balloux, 2010), implemented in the R package *adegenet*, using 1–20 assumed clusters (*K*). The number of putative clusters with the lowest Bayesian information criterion value was chosen to evaluate population groupings. Another DAPC analysis was conducted with samples taken from Canadian rivers excluded, using the same methods described above.

### 2.6 Assessing the power of SNPs and reference pool for population assignment

We tested whether our SNP panel could accurately assign individuals to populations of origin using a leave‐one‐out protocol implemented in GeneClass2 v. 2.0 (Piry et al., 2004), using the Rannala & Mountain, 1997 Bayesian method (Rannala & Mountain, 1997). Assignment success was compared to results from another genetic assignment algorithm implemented in the R package *rubias*, again using a leave‐one‐out protocol. Using this protocol, each individual is assigned to a region using a reference panel composed of all individuals except the one being tested.

We tested assignment success of all sample locations separately, as well as assignment to pooled groups according to previous population groupings used in Gauthier et al. (2013). In both cases, we considered an individual assigned to a population if the confidence score for assignment to that population was 80% or above.

## 3 RESULTS

### 3.1 Filtering

The initial SNP catalogue contained 756,713 loci. After filtering for Ln Likelihood less than −40, the catalogue contained 670,167 loci. After filtering out loci with stack depths of less than five, more than 20% missing data, more than two alleles, and loci present in fewer than 17 populations, the SNP catalogue contained 7,884 loci. After filtering by *F*_IS_ values, removing loci with minor allele frequencies <0.01 and loci in linkage disequilibrium with at least one other locus, and removing non‐polymorphic loci, we had 1,291 loci. Average read depth across loci for each individual was 55 (range = 9–171), and average read depth for loci across all individuals was 55 (range = 17–131). Sibship analysis found two possible full sibship pairs in the Kennebec River and three in the Chesapeake Bay; one individual from each pair was removed. In addition, the Cape Fear dataset contained three possible full sibship pairs, one trio, and one group of five individuals. One individual from each group was retained, and the rest were excluded from downstream analyses.

Expected and observed heterozygosity levels ranged from 0.25 to 0.38 and observed heterozygosity did not deviate more than 0.02 from expected heterozygosity in any location. Canadian rivers had slightly lower observed heterozygosity (*H*_O_ = 0.26–0.33) compared to rivers south of the Bay of Fundy (*H*_O_ = 0.35–0.38; Table 2). Similarly, individuals in the Bras d’Or–Miramichi and Shubenacadie River populations had the lowest proportion of polymorphic loci among all locations; almost one quarter of all loci genotyped were fixed in Bras d’Or–Miramichi individuals (Table 2). All other sampled locations had > 95% polymorphic loci.

**TABLE 2:** Table shows summary statistics of Striped Bass samples from 15 locations amplified at 1,291 SNP loci. Values obtained when all samples are included are in brackets

Location	*n*	# poly. loci	% poly.	Ho	He
BD‐MICHI	19	987	0.76	0.26	0.25
MIRA	22	1,234	0.96	0.27	0.29
SHUB	33	1,126	0.87	0.28	0.28
SJR	32	1,271	0.98	0.33	0.32
KEN	16	1,281	0.99	0.36	0.35
HUD 2012	34	1,289	1.00	0.36	0.36
HUD 2014	21	1,282	0.99	0.37	0.36
DEL 2012	28	1,287	1.00	0.36	0.36
DEL 2014	29	1,289	1.00	0.37	0.36
CHPK	27	1,286	1.00	0.38	0.36
POT	33	1,289	1.00	0.36	0.36
RAPP	32	1,288	1.00	0.38	0.36
JAMES	33	1,290	1.00	0.37	0.36
CHOP	33	1,283	0.99	0.36	0.36
NANTI	33	1,285	1.00	0.36	0.35
ROA	30	1,288	1.00	0.37	0.37
CF	22	1,277	0.99	0.36	0.35

Abbreviations: # poly. Loci, # of loci that are polymorphic within a population; % poly., proportion of loci that are polymorphic in a population; CF, Cape Fear; CHOP, Choptank River; CHPK, Upper Chesapeake Bay; DEL, Delaware River; He, expected heterozygosity. BD‐MICHI, Bras d’Or–Miramichi; Ho, observed heterozygosity; HUD, Hudson River; JAMES, James River; KEN, Kennebec River; MIRA, Mira River; N, Number of individuals; NANTI, Nanticoke River; POT, Potomac River; RAPP, Rappahannock River; ROA, Roanoke River; SHUB, Shubenacadie River; SJR, Saint John River.

### 3.2 Outliers

Outlier analyses identified 35 total outlier loci: BayeScan identified 13 loci as possible outliers, compared to 25 loci found by outFLANK, and 3 loci were identified by both analyses. All 35 potential outliers were excluded from downstream genetic structure analyses, while the three loci identified by both approaches were examined further as putative adaptive loci. These three loci were located on three different scaffolds and were given names according to their scaffold number and base pair position on the scaffold (scaffold_bp). Locus 4437_41108 is located 41,108 base pairs into a large (77,288 bp) scaffold, Msax_4437, inside an intron of insulin‐like growth factor 2b *(igf2b)*. The remaining two outliers, 25891_222 and 27535_2519, were located on short (2,825 and 5,316 bp, respectively) scaffolds with no known genes.

**FIGURE 2:**
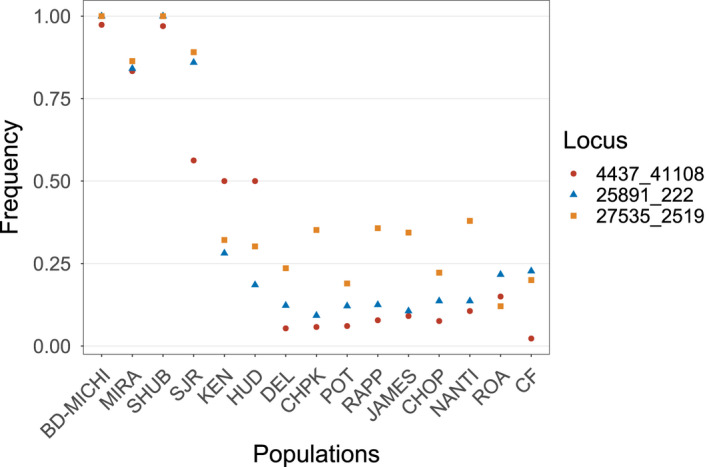
Allele frequencies of three loci identified as outliers in BayeScan and outFLANK in Striped Bass populations along the North American Atlantic Coast. The frequency of the major allele of each locus in Bras d’Or–Miramichi Striped Bass is plotted across 15 locations, displayed from north to south. BD‐MICHI, Bras d’Or–Miramichi; CF, Cape Fear; CHOP, Choptank River; CHPK, Upper Chesapeake Bay; DEL, Delaware River; HU, Hudson River; JAMES, James River; KEN, Kennebec River; MIRA, Mira River; NANTI, Nanticoke River; POT, Potomac River; RAPP, Rappahannock River; ROA, Roanoke River; SHUB, Shubenacadie River; SJR, Saint John River

Examination of allele frequencies of the three putative outliers revealed that all three loci possessed one allele that was at or near fixation in individuals within Gulf of St. Lawrence and Shubenacadie River and at very low frequencies in US locations (Figure 2), with maximum allele frequency differences of 0.85–0.98. In 25891_222 and 27535_2519, allele frequencies in Saint John River fish were slightly lower than other Canadian locations, while the major Canadian allele of 4437_41108 was present at about a frequency of 0.50 in the Saint John River, Kennebec River, and Hudson River (Figure 2).

### 3.3 Population structure

Population structure analyses were conducted using 1,256 SNPs deemed to be neutrally evolving after outlier analyses. Overall, *F*_ST_ was 0.086 and highly significant (*p* < .001), while pairwise values varied from 0 to 0.20. Correction for multiple testing did not change significance of any pairwise *F*_ST_ values. Canadian populations tended to be highly genetically distinct, while populations in the US migratory range were less genetically differentiated. The highest pairwise *F*_ST_ values occurred between Canadian rivers and all other locations (save for *F*_ST_ between the Bras d’Or–Miramichi individuals and Mira River), with *F*_ST_ values ranging from 0.09 to 0.20. Pairwise *F*_ST_ between Mira River and Bras d’Or–Miramichi River was 0.007 and nonsignificant (*p*‐value = .08; Table 3). Among the three US regions identified with genetic clustering, *F*_ST_ values ranged from 0.012 to 0.035, while within‐region *F*_ST_ values were lower (0 to 0.011). Within the Delaware River–Chesapeake Bay region, 20 of 28 comparisons were significant (Table 3). The majority (18) of significant comparisons were between James River individuals and all other locations in this region, as well as between populations in the Nanticoke and Choptank Rivers, the only two rivers sampled along the eastern side of the Chesapeake Bay, and all other locations within the region. The Delaware River, upper Chesapeake Bay, the Potomac River, and the Rappahannock River all had very low and mostly nonsignificant *F*_ST_ values with each other. The Kennebec River had very low (0.004 to 0.008) but significant (*p* < .01) pairwise *F*_ST_ compared with Hudson River individuals. Similarly, the Roanoke River and Cape Fear River had small (*F*_ST_ = 0.004) but significant differences (*p* < .001; Table 3).

**TABLE 3:** Pairwise *F*_ST_ comparisons of Striped Bass samples from 15 locations using 1,256 putatively neutral SNP loci, with significance calculated using a chi‐square test

	MIRAMICHI	MIRA	SHUB	SJR	KEN	HUD 2012	HUD 2014	DEL 2012	DEL 2014	CHPK	POT	RAPP	JAMES	CHOP	NANTI	ROA	CF
BD‐MICHI	*																
MIRA	0.007	*															
SHUB	0.201**	0.164**	*														
SJR	0.179**	0.134**	0.127**	*													
KEN	0.186**	0.132**	0.149**	0.091**	*												
HUD 2012	0.179**	0.130**	0.153**	0.094**	0.008*	*											
HUD 2014	0.183**	0.131**	0.156**	0.092**	0.004**	0.000	*										
DEL 2012	0.187**	0.134**	0.160**	0.095**	0.014**	0.015**	0.012**	*									
DEL 2014	0.182**	0.129**	0.154**	0.093**	0.012**	0.017**	0.014**	0.001	*								
CHPK	0.188**	0.133**	0.161**	0.097**	0.014**	0.017**	0.013**	0.000	0.000	*							
POT	0.179**	0.127**	0.154**	0.093**	0.013**	0.014**	0.011**	0.002*	0.000	0.002*	*						
RAPP	0.182**	0.130**	0.155**	0.094**	0.015**	0.018**	0.014**	0.000	0.001	0.000	0.002*	*					
JAMES	0.183**	0.131**	0.156**	0.098**	0.013**	0.015**	0.011**	0.003**	0.003**	0.004**	0.001*	0.004**	*				
CHOP	0.187**	0.134**	0.161**	0.097**	0.020**	0.024**	0.018**	0.002*	0.003**	0.002*	0.008**	0.001*	0.010**	*			
NANTI	0.191**	0.137**	0.161**	0.100**	0.022**	0.024**	0.021**	0.003**	0.004**	0.002*	0.008**	0.002*	0.011**	0.000	*		
ROA	0.187**	0.136**	0.155**	0.099**	0.028**	0.027**	0.027**	0.022**	0.022**	0.025**	0.021**	0.024**	0.020**	0.029**	0.029**	*	
CF	0.193**	0.140**	0.161**	0.101**	0.031**	0.029**	0.027**	0.027**	0.025**	0.028**	0.025**	0.028**	0.025**	0.034**	0.035**	0.004**	*

Abbreviations: BD‐MICHI, Bras d’Or–Miramichi; CF, Cape Fear; CHOP, Choptank River; CHPK, Upper Chesapeake Bay; DEL, Delaware River; HUD, Hudson River; JAMES, James River; KEN, Kennebec River; MIRA, Mira River; NANTI, Nanticoke River; POT, Potomac River; RAPP, Rappahannock River; ROA, Roanoke River; SHUB, Shubenacadie River; SJR, Saint John River.

* *p*‐value <.05.

** *p*‐value <.001.

**FIGURE 3:**
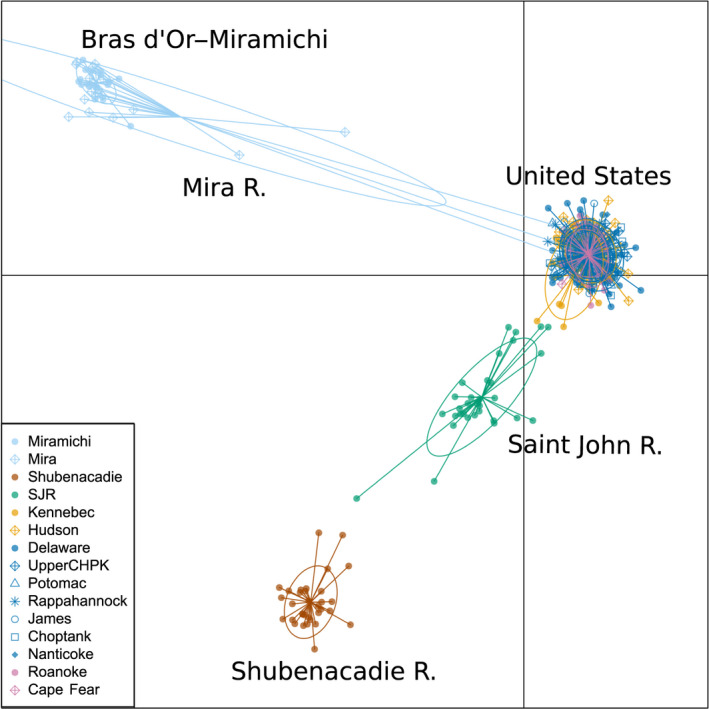
DAPC plot of Striped Bass, Morone saxatilis, populations collected in 15 locations, constructed using 1,256 SNPs. Individual Striped Bass are represented by symbols depicted in the legend, and a line connects the dot to the site it was sampled in. Distance between dots corresponds to genetic distance along two discriminant functions. Major groupings are labeled according to which populations are contained within

**FIGURE 4:**
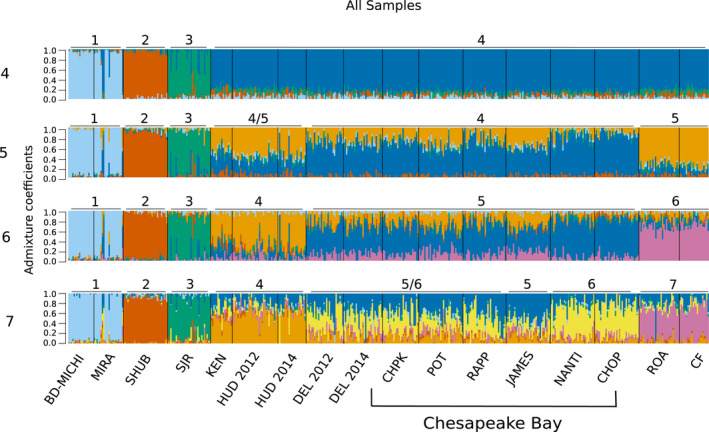
Individual admixture coefficients of Striped Bass in each site to 4, 5, 6, and 7 genetic clusters. Individual Striped Bass are represented by vertical bars, with percent genotype similarity to each cluster represented by colors. Clusters are numbered, and populations are labeled with the cluster they most resemble. Population shorthands are as follows: BD‐MICHI, Bras d’Or–Miramichi; CF, Cape Fear; CHOP, Choptank River; CHPK, Upper Chesapeake Bay; DEL, Delaware River; HUD, Hudson River; JAMES, James River; KEN, Kennebec River; MIRA, Mira River; NANTI, Nanticoke River; POT, Potomac River; RAPP, Rappahannock River; ROA, Roanoke River; SHUB, Shubenacadie River; SJR, Saint John River

Isolation‐by‐distance (IBD) and differentiation patterns differed among Canadian and US locations. Significant isolation by distance was found when all locations were considered (*r* = .84, *p* < .001). Within Chesapeake Bay, there was no significant isolation‐by‐distance pattern (*r* < .001, *p* = .50); however, when US locations in North Carolina, Hudson River, and Kennebec River were included, IBD became significant (*r* = .61, *p* = .03). When only Canadian populations were considered, there was no significant IBD (*r* = .49, *p* = .21). When all samples were run using DAPC, the most likely number of clusters was four, as estimated using the Bayesian information criterion (Figure S1). Canadian Striped Bass formed three groups, and all US Striped Bass were assigned to a fourth group (Figure 3; Table 4). This general pattern was seen when DAPC was run assuming five and six genetic groups (Figure S2). Using LEA, the number of genetic clusters (*K*) with the lowest entropy across 10 runs was 6 (Figure S1). We visualized clustering patterns for K values 4 through 7 to identify hierarchal clustering patterns as *K* increases (Figure 4). In all simulations, Canadian Striped Bass clustered into the same three groups as in DAPC. North Carolina rivers separated into their own cluster at *K* = 5, while Kennebec River and Hudson River separated at *K* = 6, and at *K* = 7, the two rivers on the eastern coast of Chesapeake Bay (Nanticoke River and Choptank River) primarily belong to the seventh cluster (Figure 4). The same clustering pattern was seen when US samples were analyzed separately from Canadian samples (Figures S3 and S4). When LEA was run with balanced sampling numbers, the lowest entropy was *K* = 4 as seen in DAPC analyses. Canadian locations clustered into three regions, while all US Striped Bass were clustered together. Mean assignment per location remained high when K was increased to 6, with the same clustering pattern seen in the full dataset (Figure S3).

**TABLE 4:** Mean ancestry coefficients of Striped Bass from 15 locations to 6 genetic clusters identified by LEA, using 1,256 putatively neutral SNP loci

	Cluster 1	Cluster 2	Cluster 3	Cluster 4	Cluster 5	Cluster 6
BD‐MICHI	0.94	0.01	0.01	0.01	0.01	0.01
Mira River	0.79	0.02	0.02	0.06	0.1	0.02
Shubenacadie	0.03	0.9	0.03	0.02	0.01	0.01
SJR	0.02	0.05	0.79	0.03	0.08	0.02
Kennebec	0.04	0.09	0.05	0.52	0.23	0.07
Hudson12	0.04	0.05	0.04	0.67	0.12	0.08
Hudson14	0.04	0.04	0.04	0.66	0.16	0.05
Delaware12	0.04	0.03	0.04	0.19	0.59	0.12
Delaware14	0.04	0.05	0.03	0.19	0.55	0.14
Chesapeake	0.04	0.03	0.03	0.19	0.61	0.1
Potomac	0.04	0.03	0.05	0.25	0.47	0.16
Rappahannock	0.03	0.03	0.03	0.16	0.64	0.1
James	0.04	0.03	0.02	0.26	0.46	0.19
Choptank	0.05	0.04	0.05	0.09	0.73	0.05
Nanticoke	0.03	0.05	0.03	0.05	0.75	0.08
Roanoke	0.03	0.04	0.04	0.1	0.12	0.68
Cape Fear	0.03	0.03	0.03	0.09	0.08	0.73

Due to the genetic distinctness of Canadian Striped Bass, it was possible to identify both putative migrants and admixed individuals within these populations. Admixture was seen in a single individual in the Mira River and eight individuals in the Saint John River. Admixed individuals within the Saint John River had approximately equal assignment to the Saint John River cluster and either the Chesapeake Bay or Shubenacadie clusters. The single admixed individual in the Mira River had equal assignment to the Bras d’Or–Miramichi cluster and to both the Hudson River and Chesapeake Bay. Additionally, three individuals caught in the Mira River appear to be migrants from Hudson River or Chesapeake Bay. When migrants and admixed individuals were removed from Mira River, both observed and expected heterozygosity for this population became 0.25, and the proportion of polymorphic loci dropped to 75% from 96%. Genetic variation within the Saint John River population also lowered slightly with the removal of admixed individuals: Observed and expected heterozygosity became 0.31, and percent polymorphism became 94% from 98%. Significance of pairwise *F*_ST_ values remained the same with and without migrants and admixed individuals. *F*_ST_ between Mira River and Bras d’Or–Miramichi Striped Bass became 0.001.

### 3.4 Assignment

Analyses were performed at two spatial resolutions to determine the geographic scale to which reliably natal assignments could be made. When individuals were compared to all 15 collection locations in GeneClass 2, 53% were assigned back to their collection location (Table S1). Of the remaining individuals, 71% were assigned to a different river including 91% of Mira River Striped Bass assigned to Bras d’Or–Miramichi, 59% of Cape Fear River Striped Bass assigned to the Roanoke River, and 48% of Striped Bass from the Delaware River and the Chesapeake Bay assigned to a different river in that area. Assignment patterns seen in North Carolina and Delaware River–Chesapeake Bay correspond to the geographic regions used in previous studies, which grouped North Carolina rivers together, and the Delaware River with all Chesapeake Bay rivers (Gauthier et al., 2013). Assignment success and accuracy were similar when analyzed with *rubias*, with 51% of individuals assigned back to their collection location and 76% of the remaining individuals assigned with high likelihood to a different river.

In the second analysis, Striped Bass were pooled into six geographic regions or proposed reporting groups: the Gulf of St. Lawrence, Shubenacadie River, Saint John River, Kennebec–Hudson River, Delaware–Chesapeake Bay, and Roanoke–Cape Fear. The three regions containing US Striped Bass correlate with groupings made by Gauthier et al. (2013), and all six regions correspond to one of the six genetic clusters identified in LEA. Under this scenario, both GeneClass2 and *rubias* assigned 99% of individuals to a reporting group: 97% to the group from which it was collected, and 2% to a different group (Table 5).

**TABLE 5:** Self‐assignment of Striped Bass samples from 6 regions (proposed reporting groups) in GeneClass2 using 1,256 putatively neutral SNP loci. Individuals were considered to belong to a reporting group if they were assigned with a confidence score of 80% or more. Rows correspond to the location individuals were collected in, while columns correspond to assigned reporting group

Location	GoSL	SHUB	SJR	KEN‐HUD	DEL‐CHPK	N. Carolina	Unassigned
GoSL	37				4		
SHUB		33					
SJR			28		2		2
KEN‐HUD				65	4	1	1
DEL‐CHPK					247		1
ROA‐CF					1	51	

Abbreviations: DEL‐CHPK, Delaware River and all rivers within Chesapeake Bay; GoSL, Gulf of St. Lawrence; KEN‐HUD, Kennebec River and Hudson River; ROA‐CF, Roanoke River and Cape Fear River; SHUB, Shubenacadie River; SJR, Saint John River.

## 4 DISCUSSION

We present the most complete examination of Striped Bass genetic structure across their native range using SNP loci. Previous genetic studies of Striped Bass have used genetic markers such as RFLPs (e.g., Wirgin, Maceda, Stabile, & Mesing, 1997; Wirgin, Maceda, et al., 1993), VNTRs (Laughlin & Turner, 1996), mitochondrial sequences (e.g., Wirgin, Maceda, et al., 1997), microsatellites (Bentzen & Paterson, 2008; Brown et al., 2005; Gauthier et al., 2013; Wirgin et al., 2020), and SNP loci (LeBlanc et al., 2018) to assess the genetic structure of Striped Bass across portions of its range. Only two studies have included a thorough coverage of all major migratory populations (Hudson River to Roanoke River), and of those only Wirgin et al. (2020) has included Canadian populations. In addition, this is the first published study to include samples from Mira River, which hosts a largely unknown group of Striped Bass that may support a spawning aggregation (Andrews, Dadswell, et al., 2019; Buhariwalla, 2018), and to document the presence of US origin Striped Bass on the northeastern coast of Nova Scotia. Our study found significant genetic structure partitioned into six regions, and much greater differentiation of Canadian regions from each other and all US regions. Our SNP panel was able to assign Striped Bass to one of these six regions with a 99% success rate. We also identified three SNP loci that show signs of selection across the sampled Striped Bass range.

### 4.1 Genetic diversity

Genetic diversity was highest among sampled US locations and lowest in the relatively isolated populations in the Gulf of St. Lawrence and the Shubenacadie River. All sampled US populations had similar mean observed heterozygosity, including the small and hatchery‐supported Cape Fear River, suggesting that genetic variation comparable to the rest of the US range is being maintained in this population. The Kennebec River population similarly does not show signs of reduced genetic diversity, despite the Kennebec River's recent restoration with stocked Hudson River Striped Bass (G. Whippelhauser, pers. comm.). Within Canada, the highest observed heterozygosity was seen in the Saint John River, only slightly lower than values seen in the United States, suggesting this population has retained substantial genetic diversity despite its apparent decline in numbers since the 1970s (Andrews et al., 2017; LeBlanc et al., 2018). Diversity values calculated for all populations were comparable to patterns seen in a recent range‐wise microsatellite study (Wirgin et al., 2020). Lower genetic diversity in northern, previously glaciated locations has been seen in other anadromous fish species such as American Shad (*Alosa sapidissima*; Hasselman, Ricard, & Bentzen, 2013) and is expected in populations on the edge of a range expansion (Bernatchez & Wilson, 1998; Hewitt, 1996). The lowest observed heterozygosity values seen in this study (0.26–0.28) were similar to observed heterozygosity seen in other anadromous fishes examined using SNP markers, such as Blueback Herring (*H*o = 0.28–0.30), Alewife (*H*o = 0.22–0.27; Baetscher et al., 2017), and Chinook Salmon (*Oncorhynchus tshawytscha;* *H*o = 0.26–0.32; (Clemento, Abadía‐Cardoso, Starks, & Garza, 2011).

### 4.2 Outlier loci represent regions of major effect

Most ecologically relevant traits are thought to be polygenic, involving small allele frequency differences of many genes (Pavey et al., 2015; Yeaman, 2015). All three outliers identified in this study showed high allele frequency changes among populations (0.85–0.97 maximum allele frequency differences), which suggests the presence of single genes or regions of major effect. The two unannotated outlier loci identified in this study (25891_222 and 27535_2519) showed a strong tendency toward fixation for one allele in Canadian populations, and very low frequency of that allele in southern populations, while locus 4437_41108 showed a tendency toward fixation of allele *A* in Shubenacadie River and Gulf of St. Lawrence Striped Bass and very high allele frequencies of allele *B* in populations south of the Hudson River. Within Striped Bass in the Saint John River, Kennebec River, and Hudson River, in contrast, both alleles were maintained at relatively equal frequency. Further characterization of the Striped Bass genome and anchoring of existing scaffolds into linkage groups will be invaluable for placing these outlier loci into a wider genomic context, and sequencing of *igf2b* in northern versus southern individuals will shed light on whether locus 4437_41108 is associated with nonsynonymous mutations within this gene.

### 4.3 Canadian populations are highly distinct

Rivers near the Gulf of St. Lawrence (Bras d’Or–Miramichi and Mira River), the Shubenacadie River, and the Saint John River were consistently, highly differentiated from each other and from US populations (*F*_ST_ = 0.13–0.20). Phylogeographic theory predicts that populations founded after the last glacial retreat will show less intraspecific divergence than their southern counterparts (Bernatchez & Wilson, 1998). Unexpectedly high divergence in Canadian populations has been seen in other anadromous fishes along the North American Atlantic coast and has been attributed both to the circuitous coastline created by the Nova Scotia peninsula and to a complex hydrography within the Bay of Fundy that drives differentiation of native fish populations (Hasselman et al., 2013; King, Kalinowski, Schill, Spidle, & Lubinski, 2001; McConnell, Ruzzante, O’Reilly, Hamilton, & Wright, 1997). Variation in habitat is known to drive differentiation of anadromous fish species such as Atlantic Salmon (Bradbury et al., 2014) and Dolly Varden Char (*Salvelinus malma*; Bond, Crane, Larson, & Quinn, 2014). The Shubenacadie River, in particular, is the only tidal bore river wherein Striped Bass are known to successfully spawn (Rulifson & Dadswell, 1995), and the extreme environmental conditions that eggs and larvae must tolerate in this river may contribute to its increased population differentiation (Rulifson & Tull, 1999). Unexpectedly high genetic divergence in Canadian populations could also be the result of small initial colonization sizes driving changes in allele frequencies that persist to the present day (Excoffier & Ray, 2008).

Genetic similarity between the Mira River and Bras d’Or–Miramichi Striped Bass indicates that these two groups have the same origin. It is likely that Striped Bass currently residing in the Mira River migrated from the Miramichi River at some point after the formation of suitable estuarine habitat and nursery areas some 500–800 years ago (Andrews, Dadswell, et al., 2019). While Striped Bass in the Mira River appear behaviorally distinct, demonstrating multiannual residency and spring upstream migration shown in an acoustic telemetry study in 2012–2015 (Andrews, Dadswell, et al., 2019; Buhariwalla, 2018), our data suggest that this potential spawning population is not genetically distinct from Striped Bass found within the Gulf of St. Lawrence.

Also identified from Mira River samples were individuals of putative US origin (3/22 samples). Recent evidence that Striped Bass move between the US Atlantic coast and the northeastern coast of Nova Scotia is scarce. In 1983, a single fish tagged in the Kouchibouguac River in the Gulf of St. Lawrence in 1983 was later recaptured in the Wye River, Maryland (Hogans & Melvin, 1984), indicating that this fish likely passed through the northeastern coast of Nova Scotia. In contrast, none of the hundreds of Striped Bass with internal acoustic tags in the Roanoke River, Hudson River, New England coast, Bay of Fundy, and Miramichi River (Andrews, Wallace, Gautreau, Linnansaari, & Curry, 2018; Broome, 2014; Callihan et al., 2015; Douglas, Bradford, & Chaput, 2003; Gahagan et al., 2015; Pautzke, Mather, Finn, Deegan, & Muth, 2010) have ever been detected passing the Halifax Line of acoustic receivers on the eastern coast of Nova Scotia (Andrews, Dadswell, et al., 2019). Thousands of Striped Bass in the Gulf of St. Lawrence (DFO, 2010; Douglas et al., 2003; Hogans & Melvin, 1984), the Bay of Fundy (Broome, 2014; Rulifson & Dadswell, 1995), and along the US coast (Pautzke et al., 2010; Richards & Rago, 1999; Waldman et al., 1990) have been externally tagged from the 1960s to the present day (Andrews, Dadswell, et al., 2019), only one of which has ever been caught on the far eastern shores of Nova Scotia (Andrews et al., 2018; Douglas et al., 2003). This apparent isolation may be caused by a physical isolation of the Gulf of St. Lawrence before the Canso Strait opened postglacier retreat (Shaw & Courtney, 2002) and after the Canso Causeway was built in 1955 (Vilks, Schafer, & Walker, 1975), or influenced by a sharp temperature change between the two water bodies (Rulifson & Dadswell, 1995). A “genetic breakpoint” has been described in several other species along eastern Nova Scotia at ~45°*N* (close to the City of Halifax; Stanley et al., 2018). Increasing ocean temperatures are predicted to drive Striped Bass populations north, but this remains a poorly studied region.

The presence of a genetically distinct population of Striped Bass in the Saint John River following its suggested extirpation in the 1970s has been debated for over a decade (Andrews et al., 2017). Two previous studies have found evidence of unique genotypes distinct from US and Shubenacadie River Striped Bass, and present in adults (Bentzen & Paterson, 2008) and juveniles (LeBlanc et al., 2018). A third study examined a mixture of 17 juveniles and 25 adults collected from the Saint John River in 2014 and found that all fish showed admixture between Shubenacadie River and US genotypes with no unique cluster (Wirgin et al., 2020). The 17 juveniles examined by Wirgin et al. (2020) are included in this present study, along with 15 additional juveniles collected in 2015–2017. In contrast to Wirgin 2020s results, most juveniles we examined showed a distinct genetic signature and admixture was only seen between the Saint John River cluster and either Shubenacadie or US genotypes. We detected no Shubenacadie–US hybrids. Adult and juvenile Saint John River Striped Bass examined in both studies are also part of an ongoing (6 + year) acoustic telemetry study. Initial telemetry results clearly demonstrate differing migratory patterns between adults genotyped as Shubenacadie origin (which left the river to spawn), adults genotyped as belonging to the Saint John River cluster (which migrated upstream) and those of US origin Striped Bass (which aggregated around the Hammond River, a tributary of the Saint John River; Andrews, Linnansaari, et al., 2019).

Striped Bass from US populations have been found in Minas Basin (Bay of Fundy; Rulifson, McKenna, & Dadswell, 2008) and are thought to enter the Shubenacadie River as well, and this study found no evidence that migrants successfully spawn in the Shubenacadie River. In contrast, juveniles from the Saint John River were admixed with Shubenacadie River and Chesapeake Bay populations. It is unknown how often this gene flow occurs now or prior to the population's apparent disappearance in the 1970s. All admixed juveniles we examined had approximately equal assignment to the Saint John River and the Shubenacadie River/Chesapeake Bay clusters, suggesting intraspecific F1 hybrids. The first‐generation hybrids and the distinctness of the Saint John River–US genotypes suggest that these admixed juveniles may be a recent development. There is little information about the proportion of US migrants in the Saint John River before the population crash and no information about possible admixed individuals (Andrews et al., 2017). Larger Striped Bass are more likely to migrate and to travel far (Andrews, Dadswell, et al., 2019; Callihan et al., 2014; DFO, 2018), and as Striped Bass populations recover, there is an increase in the number of older, larger individuals making migrations (Callihan et al., 2014). We hypothesize that the admixed juveniles result from small numbers of local spawners making admixed offspring more prevalent, increased migration from recovering populations, and a climate‐induced northward range shift.

### 4.4 US Regional Structure

In contrast to Canadian Striped Bass, *F*_ST_ values among US locations were much lower (*F*_ST_ = 0.000–0.035) and support three regions with low but significant genetic divergence: (a) Hudson River and Kennebec River, (b) Delaware River and Chesapeake Bay, and (c) North Carolina Rivers. Overall, our results suggest individual spawning populations within the Delaware River and Chesapeake Bay make up a large metapopulation connected by extensive gene flow, with lesser amounts of gene flow between this region and populations to the north and south.

Connectivity among populations of Striped Bass along the Atlantic Coast has been investigated in several previous studies (Able, Grothues, Turnure, Byrne, & Clerkin, 2012; Bentzen & Paterson, 2008; Brown et al., 2005; Callihan et al., 2015; Gauthier et al., 2013) and is influenced by gene flow, stocking, and possibly recolonization following local extirpation. Striped Bass between Maine and North Carolina are highly migratory (>1,000 km; Callihan et al., 2015), compared to the more restricted migratory range of populations in Canada and the apparent complete residency of populations south of Roanoke River, North Carolina. Several tagging studies have previously documented movement of Striped Bass among Chesapeake Bay, Kennebec River, Hudson River, and Roanoke River (Callihan et al., 2015; Dorazio, Hattala, McCollough, & Skjeveland, 1994; Gahagan et al., 2015; Kneebone et al., 2014; Waldman et al., 1990). The presence of an isolation‐by‐distance pattern of differentiation among US locations but not among Canadian locations further supports gene flow among populations in this range.

Our study also investigates the current genetic profile of the recently restored Kennebec River population of Striped Bass. The Kennebec River is one of several rivers in Maine that likely once hosted a native population of Striped Bass (Little, 1995). This population declined and was likely extirpated by the late 1930s, and was subsequently stocked with over 260,000 Striped Bass juveniles from 1982 to 1991 from the Hudson River in an attempt to restore the population (G. Whippelhauser, pers. comm.). Considering stocking, it is unsurprising that the juvenile Striped Bass caught in the Kennebec River in this study were most similar to the Hudson River. *F*_ST_ values between the two rivers were low (*F*_ST_ = 0.008) but significant, and similar to values seen among some rivers within the Chesapeake Bay, indicating a similar level of relatedness despite the large geographic distance between them (approximately 620 km from mouth to mouth). This similarity was also seen in a recent microsatellite study that examined Kennebec River juveniles (Wirgin et al., 2020), which found no statistically significant difference between the Kennebec River and Hudson River.

Within the Chesapeake Bay and Delaware River, *F*_ST_ values were very low. The highest values were seen when comparing James River individuals to other rivers in the Bay, as well as Nanticoke and Choptank Rivers, both located on the east coast of the Bay, to rivers on the west coast. A small amount of differentiation between the east and west coast of Chesapeake Bay was also seen in the most recent microsatellite study done on Striped Bass (Wirgin et al., 2020) and may be due to the channel of deeper water that runs through the center of the Bay. The lowest *F*_ST_ values within the Chesapeake–Delaware region were seen between individuals from the head of Chesapeake Bay and Delaware River, supporting the hypothesis that these two groups of Striped Bass are not genetically distinct from one another. Previous genetic studies have found conflicting results on whether the growing Striped Bass population in the Delaware was distinct from the Chesapeake Bay. An analysis of mitochondrial length–frequency differences in the recovered Delaware River Striped Bass found significant differences in minor length–frequency alleles from Chesapeake Bay Striped Bass (Waldman & Wirgin, 1994). Minor length–frequency differences were also seen among tributaries within the Chesapeake Bay (Wirgin, Maceda, et al., 1993), and microsatellite studies which found significant *F*_ST_ values between the Delaware River and the Chesapeake Bay also found *F*_ST_ values of the same magnitude among tributaries within the bay (Gauthier et al., 2013). Decades of observations of adult Striped Bass using the Chesapeake and Delaware Canal to transit between the Chesapeake and Delaware estuaries during spawning season (Kneebone et al., 2014; Koo & Wilson, 1972; Nichols & Miller, 1967) support the likelihood that the Delaware River population receives a high amount of gene flow from Chesapeake Bay Striped Bass, on a similar magnitude as seen among rivers within the Bay. Whether the current Delaware River Striped Bass are descended from a remnant population that was genetically similar to the Chesapeake Bay or whether they are descended from Chesapeake Bay Striped Bass that recolonized the river, it seems clear that Delaware River Striped Bass are part of a complex network of gene flow among the tributaries of the Chesapeake Bay.

### 4.5 Assignment

Self‐assignment tests were performed on the SNP panel generated in this study to assess its utility as a reference dataset for future mixed‐stock analyses. Previous attempts to use genetic markers for mixed‐stock analysis have met with limited success. Most recently, a study conducted self‐assignment tests using GeneClass2 on 14 microsatellites and was able to assign 60% of Striped Bass from the Hudson River, Chesapeake Bay (including the Delaware River), North Carolina, and South Carolina to a region of origin (Gauthier et al., 2013). Our SNP panel showed the highest assignment success when overlapping populations were grouped into the same reporting groups used by Gauthier et al. (2013). We were able to assign 99% of Striped Bass to a region of origin with >80% confidence. When individuals were assigned to river of origin (rather than region of origin), assignment success was much lower and individuals were misassigned to other rivers within the same region, reflecting the low genetic differentiation among these rivers. The assignment success rate seen within regions is likely an indication that rivers within a region are not demographically independent.

Statistical biases when using large panels of SNP loci have been identified in assignment tests that use simulated individuals to predict assignment accuracy of a set of loci (Anderson, Waples, & Kalinowski, 2008); however, the data in this present study indicate that self‐assignment tests in the absence of simulations can result in misleadingly high confidence values. In light of emerging techniques allowing high‐throughput genotyping of large numbers (>1,000) of loci (Ali et al., 2016), researchers looking to assess stock composition of increasingly closely related populations should interpret confidence scores with these issues in mind when choosing a geographic resolution in which to assign fish. In addition, admixed individuals seen in the Saint John River were assigned to one of their parent populations with high confidence, suggesting that assignment in both GeneClass2 and *rubias* is insensitive to the presence of admixed individuals. When performing mixed‐stock analysis on locations with large numbers of hybrid individuals, assignment may be better conducted using a genetic clustering algorithm such as those found in LEA or STRUCTURE. Overall, our SNP panel constitutes a significant improvement over other genetic markers in assigning Striped Bass to regional areas along the Atlantic coast and will be invaluable to the development of a highly accurate and reliable genetic tool for mixed‐stock analysis of the species across the central and northern portion of their range.

## 5 CONCLUSION

Striped Bass have been thought to exhibit a high degree of natal homing (Pess et al., 2014), but recent genetic and telemetry studies indicate the species expresses more variability in homing to their natal river (e.g., Callihan et al., 2015; Gahagan et al., 2015). Studies document skipped spawning and straying among populations (Gahagan et al., 2015; Kneebone et al., 2014). Low or nonexistent genetic structure among tributaries in the Chesapeake Bay and the connected Delaware River (see also Brown et al., 2005; Gauthier et al., 2013) suggests that straying or colonization among rivers in this region is common. Canadian populations at the northern range limit exhibited greater genetic isolation, but with evidence of hybridization with US individuals in the Saint John River and detection of US individuals in the Mira River. Genetic structure in the north may relate to the relatively recent opening of the rivers, that is, postglaciation about 10,00 years ago and/or more recent climatic changes and population increases pushing US migrants farther north. Regardless, the variable exchanges among rivers provide a zoogeographic dynamic with important implications for the local and international management of Striped Bass.

This study represents the first contribution that used genotyping by sequencing to facilitate highly accurate mixed‐stock analyses of Striped Bass along the Atlantic Coast, including stock compositions in the Bay of Fundy and ongoing characterization of Striped Bass along the Nova Scotian coast. This mixed‐stock analysis method will be especially valuable if climate change influences shifts in the range of Striped Bass and results in increased mixing of different spawning populations across international borders, allowing for early detection and appropriate responses in management and policy.

## CONFLICT OF INTEREST

None declared.

## Supporting information

Fig S1‐S4

Table S1

## Data Availability

Raw sequencing data are available on the US National Center for Biotechnology Information, BioProjects [PRJNA627492]. Data input files are available in the Dryad repository, https://doi.org/10.5061/dryad.9kd51c5dd.
