# Supplementary material for: Genomic population structure of Striped Bass (Morone saxatilis) from the Gulf of St. Lawrence to Cape Fear River
Source: Evol Appl. 2020 May 29;13(6):1468–86. doi: 10.1111/eva.12990 (PMC7359840; doi:10.1111/eva.12990)
Supplement: Supplementary file 1 — Fig S1‐S4 [file EVA-13-1468-s001.docx]

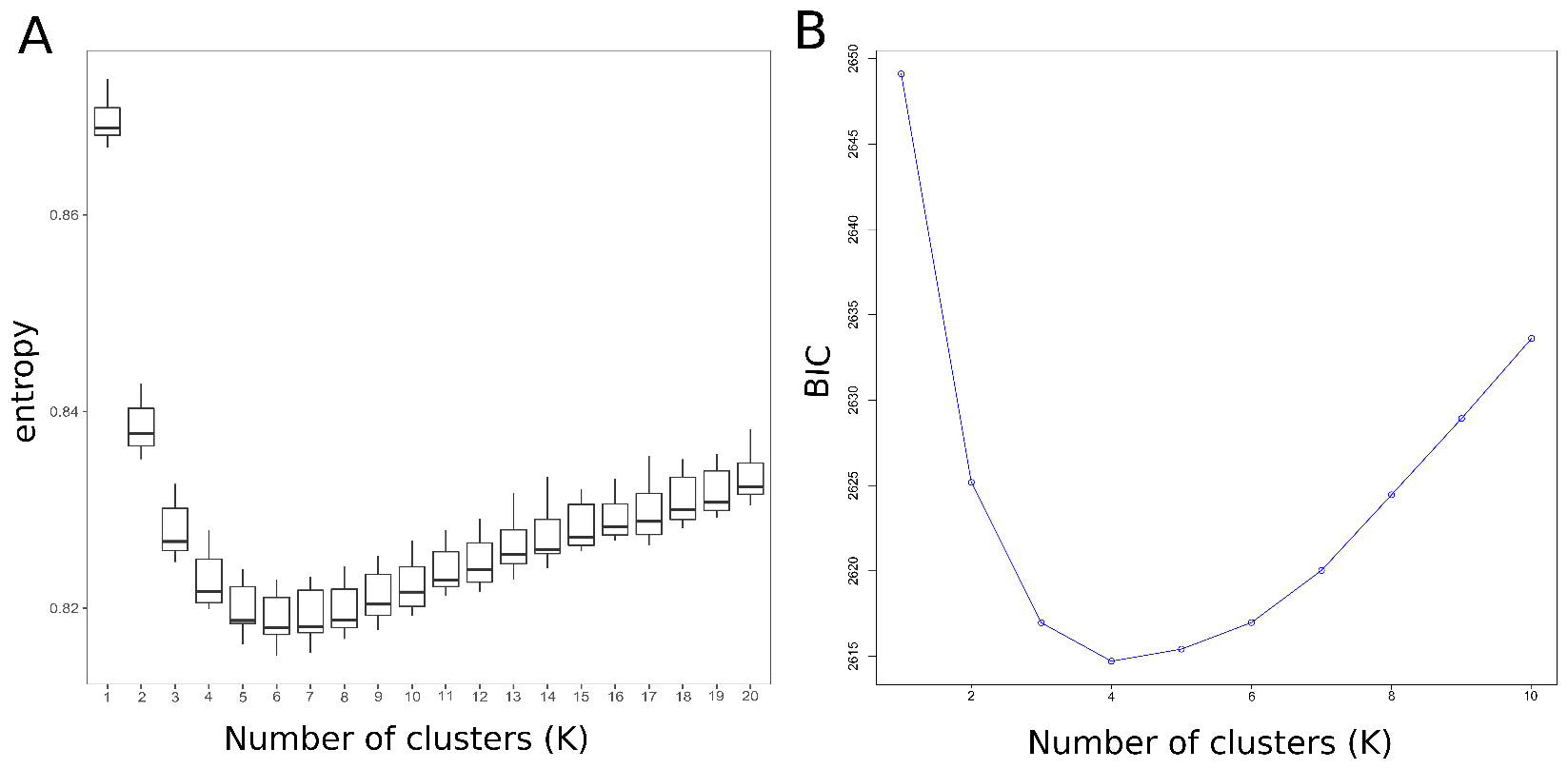


**Figure S1.** A) Entropy values calculated using the cross-entropy criterion for genetic clusters implemented in LEA, inferred from Striped Bass (*Morone saxatilis*) populations collected in 15 locations, using 1,256 putatively neutral SNP loci. B) Bayesian Information Criterion values for most likely number of clusters in DAPC, run with the same samples.


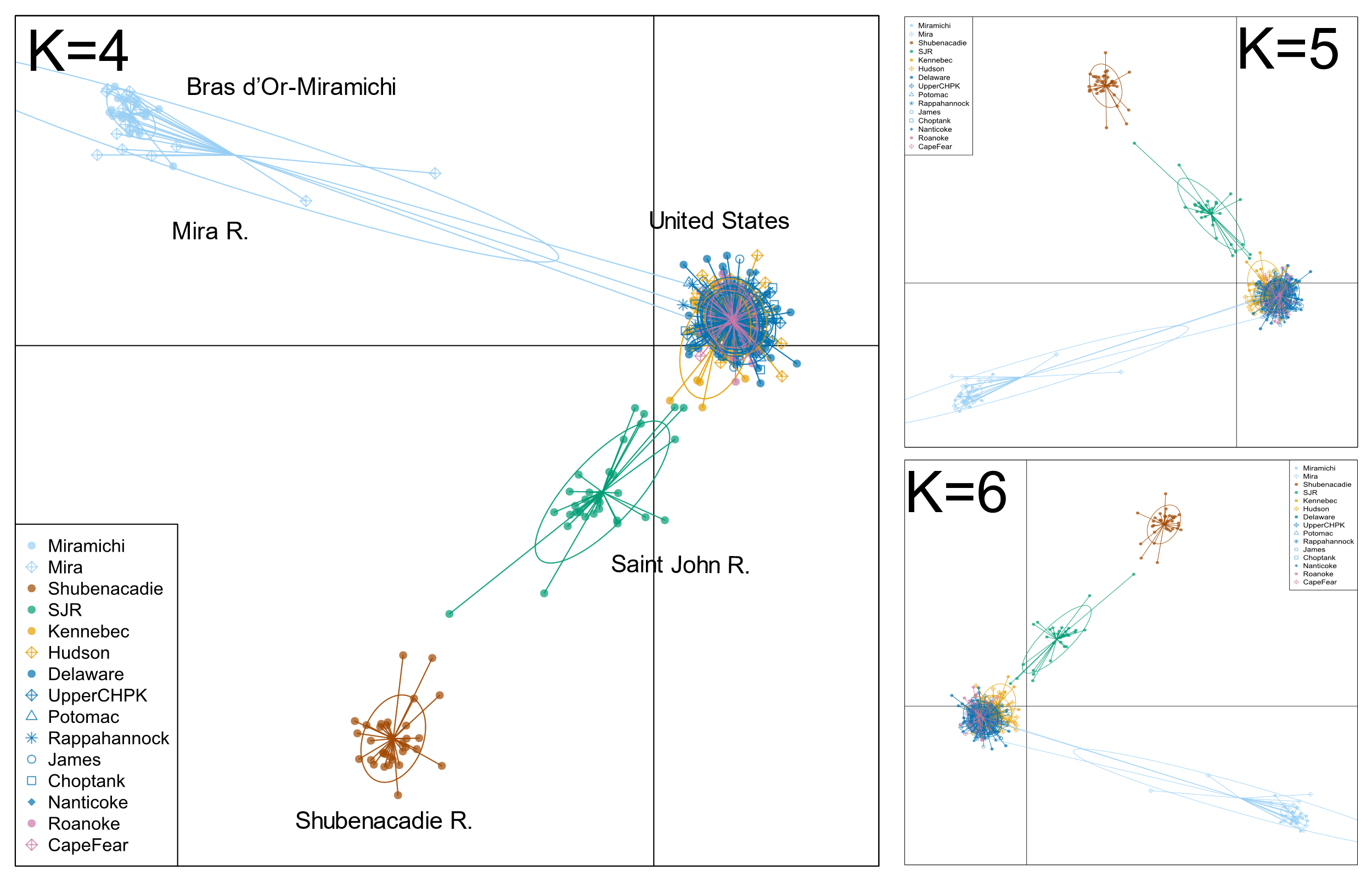


**Figure S2.** DAPC plots of Striped Bass, *Morone saxatilis*, populations collected in 15 locations, constructed using 1,256 putatively neutral SNP loci and calculated assuming 1) 4 groups, B) 5 groups, C) 6 groups. Individual Striped Bass are represented by symbols depicted in the legend, and a line connects the dot to the site it was sampled in. Distance between dots corresponds to genetic distance along two discriminant functions. Major groupings are labelled according to which populations are contained within.


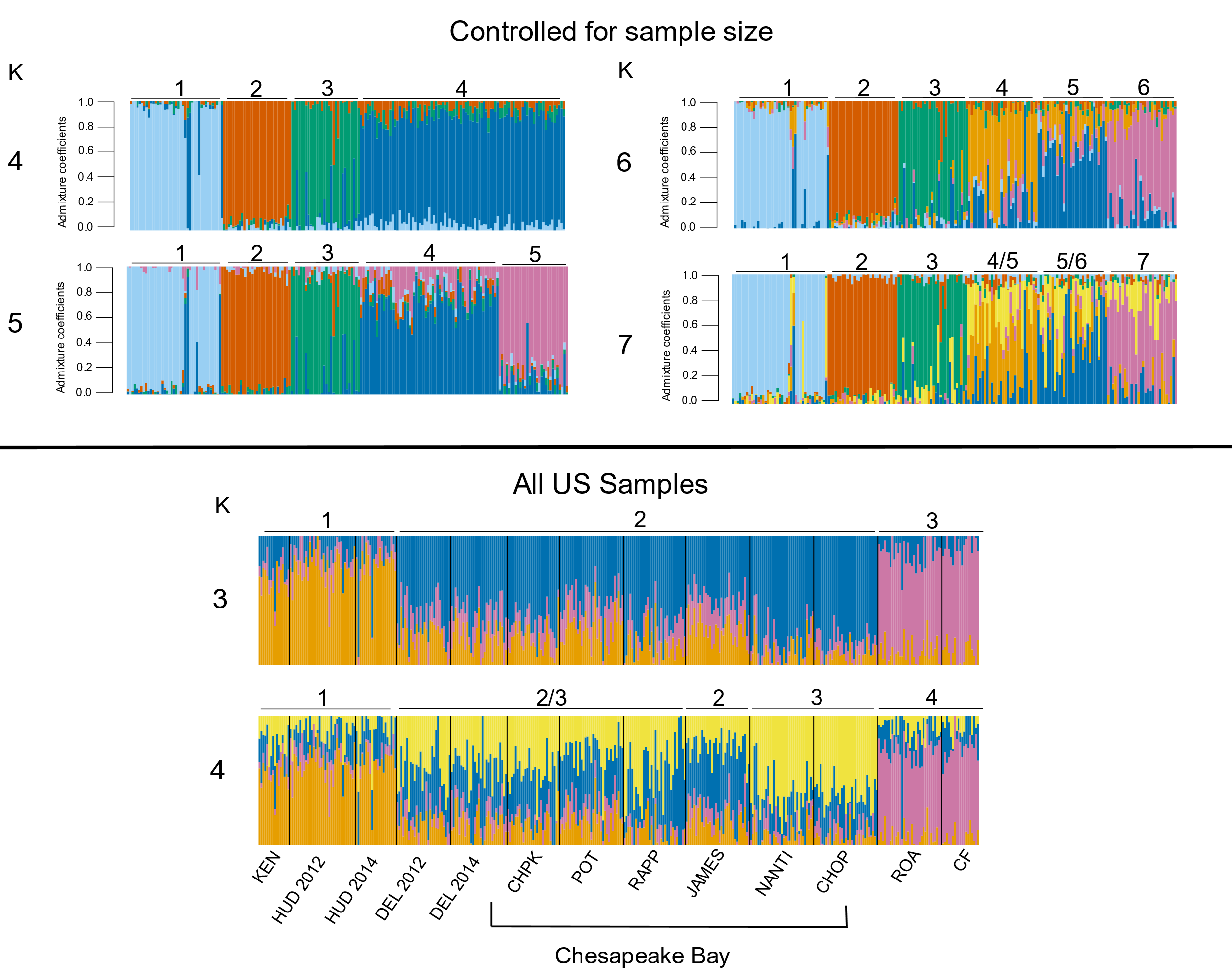


**Figure S3.** On the top, individual admixture coefficients of Striped Bass controlled for sample size, using clusters found at K=6. All results calculated using 1,256 putatively neutral SNP loci. On the bottom, individual admixture coefficients of 374 Striped Bass, *Morone saxatilis*, collected at 11 locations to 3 and 4 genetic clusters. Individual Striped Bass are represented by vertical bars, with percent genotype similarity to each cluster represented by colours. Clusters are numbered and locations are labelled with the cluster they most resemble. Population shorthands are as follows: KEN = Kennebec River, HUD = Hudson River, DEL = Delaware River, CHPK = Upper Chesapeake Bay, POT = Potomac River, RAPP = Rappahannock River, JAMES = James River, CHOP = Choptank River, NANTI = Nanticoke River, ROA = Roanoke River, CF = Cape Fear.


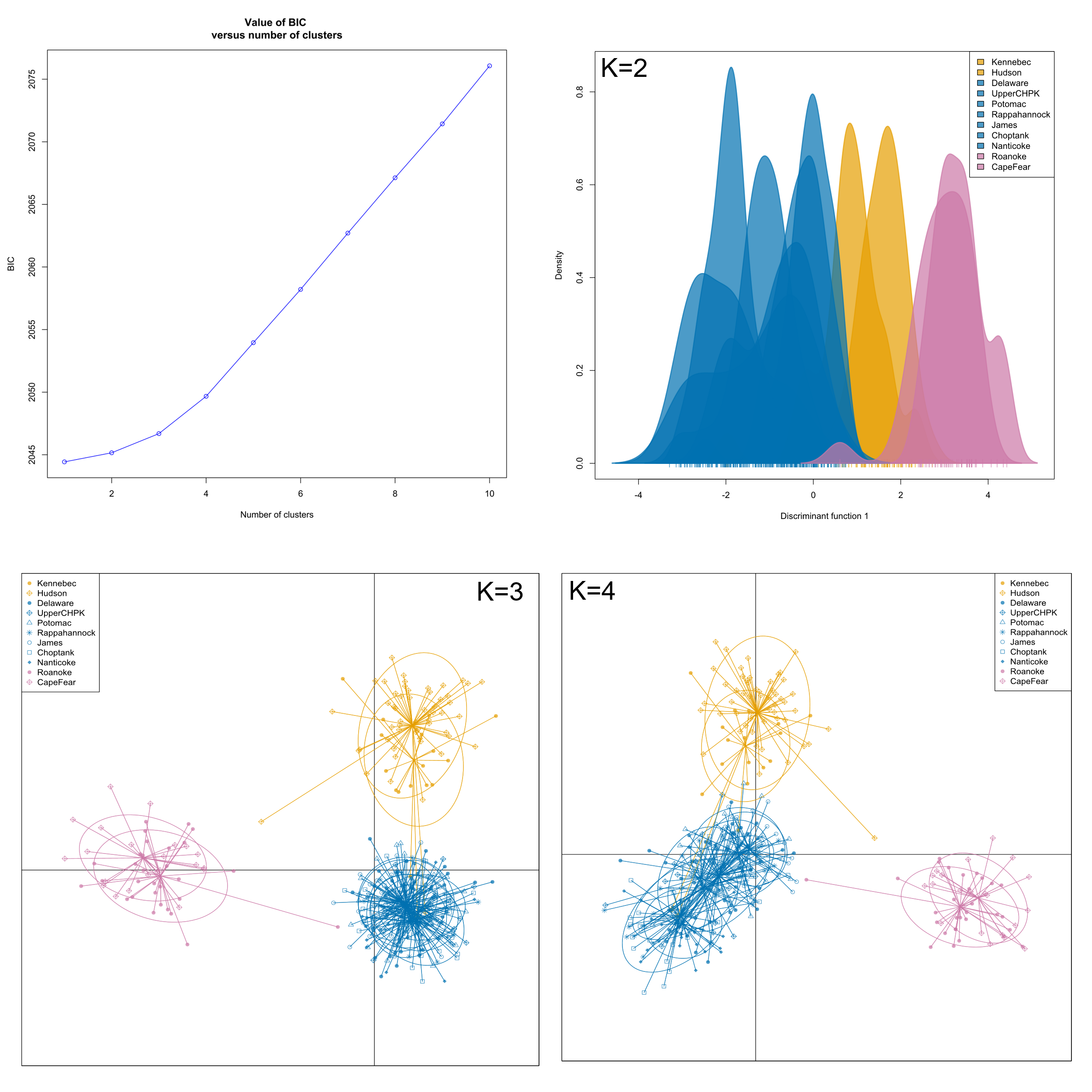


**Figure S4.** 1) Bayesian information criterion estimating the most likely number of groups from which Striped Bass collected at 11 locations come, where lower values indicate higher likelihood. 2-4) DAPC plots of Striped Bass, *Morone saxatilis*, populations collected at 11 locations, constructed using 1,256 SNPs and calculated assuming 2) 2 groups, 3) 3 groups, 4) 4 groups. Individual Striped Bass are represented by symbols depicted in the legend, and a line connects the dot to the site it was sampled in. Distance between dots corresponds to genetic distance along two discriminant functions. Major groupings are labelled according to which populations are contained within.
